# Trabecular Architecture of the Manual Elements Reflects Locomotor Patterns in Primates

**DOI:** 10.1371/journal.pone.0120436

**Published:** 2015-03-20

**Authors:** Stacey A. Matarazzo

**Affiliations:** Anthropology Department, University of Massachusetts at Amherst, Massachusetts, United States of America; University of Illinois at Urbana-Champaign, UNITED STATES

## Abstract

The morphology of trabecular bone has proven sensitive to loading patterns in the long bones and metacarpal heads of primates. It is expected that we should also see differences in the manual digits of primates that practice different methods of locomotion. Primate proximal and middle phalanges are load-bearing elements that are held in different postures and experience different mechanical strains during suspension, quadrupedalism, and knuckle walking. Micro CT scans of the middle phalanx, proximal phalanx and the metacarpal head of the third ray were used to examine the pattern of trabecular orientation in *Pan*, *Gorilla*, *Pongo*, *Hylobates* and *Macaca*. Several zones, i.e., the proximal ends of both phalanges and the metacarpal heads, were capable of distinguishing between knuckle-walking, quadrupedal, and suspensory primates. Orientation and shape seem to be the primary distinguishing factors but differences in bone volume, isotropy index, and degree of anisotropy were seen across included taxa. Suspensory primates show primarily proximodistal alignment in all zones, and quadrupeds more palmar-dorsal orientation in several zones. Knuckle walkers are characterized by having proximodistal alignment in the proximal ends of the phalanges and a palmar-dorsal alignment in the distal ends and metacarpal heads. These structural differences may be used to infer locmotor propensities of extinct primate taxa.

## Introduction

Living bone is a dynamic tissue that functionally adapts in response to mechanical loading. This functional adaptability of bone tissue, commonly referred to as "Wolff's Law", is seen in changes to the external morphology of skeletal elements, and *within* the internal trabecular structure. Although the initial mathematical tenants of Wolff’s law have been discredited, the ability of trabecular bone to align along a functional axis has been revealed in several empirical studies [1–8]. The majority of these studies seek to explore possible trabecular bone alignment differences in animals that use different locomotor and/or positional behaviors. Trabecular bone is more porous than cortical bone; a feature that allows for greater surface area and increased cellular components [3]. This increase in cells enables trabecular bone to be more metabolically active and respond to locomotor and positional loading to a potentially greater degree than the densely packed cortical bone [3]. This responsive ability makes trabecular bone an attractive feature to examine when exploring locomotor capabilities in extinct animals.

With micro Computed Tomography (μCT) (employed in the above studies), it is now possible to examine the internal structure of bone in finer detail and in three dimensions [9–15]. Micro CT scans have been used to examine changes in cortical bone morphology, trabecular structure, and subchondral bone thickness mainly in primate long bones as a means to explore locomotor differences [16–23]. The results of these analyses vary. While MacLatchy and Muller [17] and Ryan and Ketcham [19] revealed a relationship between femoral head trabecular orientation and locomotor patterns in several Strepsirrhine species, this relationship does not seem to hold true for anthropoids. Trabecular structure alone (of the humeral and femoral heads) appears similar across a wide range of anthropoid primates and does not discriminate between locomotor groups [21–22]. However, a locomotor signal is present when a *suite* of trabecular features are considered [20]. Once body size is controlled, the suite of femoral head trabecular features (trabecular number, degree of anisotropy, connectivity density, and structure model index) discriminates well among anthropoids assigned to different locomotor categories. The humeral head suite of features shows a similar but weaker locomotor signal [20]. Scherf et al. [23] also noted differences in the humeral head trabecular architecture of *Pan*, *Pongo*, and *Homo* when a suite of features is considered, and argues that a multivariate statistical analysis is needed to capture species specific combinations of trabecular bone features. They characterize the humeral trabecular bone of *Pan* as closely arranged and plate-like; those of *Pongo* as plate-like but thicker and widely spaced; and those of *H*. *sapiens* of being rod-like and fewer in number.

Although most locomotor studies have focused on the long bones, there have been several attempts to explore locomotion in the wrists and metacarpals of primates using micro CT analyses along with traditional histological preparations, morphometrics, and CT osteoabsorptiometry (OAM). Studies of the distal radius using CT-OAM revealed greater density in the subchondral bone of the ventral region in knuckle walkers that distinguishes them from humans, quadrupeds and orangutans [24–25]. Greater density in this region is expected given the enhanced loading placed upon the area while the hand is in the knuckle-walking posture (with extension at the wrist and metacarpals). In contrast, digitigrade primates who keep the metacarpals in line with the wrist during the stance phase of walking exhibit the greatest density in the central region of the distal radius [25]. When microtomographic analysis was used to examine primate carpals (scaphoid, lunate, and capitate) of *Gorilla*, *Pan*, *Pongo*, *Symphalangus*, *Hylobates*, *Papio*, and *Ateles*, no clear functional signal was seen [26]. Schilling et al. [26] noted only differences between humans and non-human primates: the non-load bearing human carpals had a significantly lower bone volume (BV/TV). Differences were noted in the metacarpals. Zeininger et al. [27] examined the third metacarpal heads of *Pan*, *Pongo*, and *Homo* using backscattered electron microscopy and revealed, that in contrast to *Pongo* and humans, *Pan* displays greater remodeling rates and loading at the dorsal and palmar regions. Cross-sectional properties of metacarpals 3 and 4 reveal greater robusticity in knuckle walkers than is seen in orangutans and humans [28]. This relationship between hand posture and metacarpal shape is also seen in cercopithecoids [29].

Micro CT studies of extant primate metacarpals have shown some inter- and intraspecific differences within hominoids. Chirchir et al. [30] found that *Pan* had greater bone density in the dorsal region of the third metacarpal head than *Pongo* and suggests this is due to greater loading of this region in chimpanzees during knuckle walking. Lazenby et al. [31] noted differences in overall robusticity of metacarpals 1, 2,and 5 between Cameroon chimpanzees and those of the Thai forest. They attribute the greater robusticity of the Cameroon chimpanzee metacarpals to more frequent knuckle walking and/or greater tool use. Further differences were noted in the trabecular bone of the metacarpal heads of humans, knuckle-walkers, and brachiators [32]. Tsegai et al. [32] applied a new method to examine trabecular structure of hominoid metacarpals which analyzed the entire epiphyseal head: (as opposed to traditional volume of interest studies which consider only a selected area of trabecular bone). Their results show trabecular bone volume distribution and areas of greatest stiffness differed between suspensory primatess, knuckle-walkers, and humans and these differences correspond with predicted loading patterns. Knuckle walkers show greater bone volume and stiffness throughout the metacarpal head.

To date very little examination of the trabecular structure of the manual phalanges has been undertaken. Preliminary analyses of the manual phalanges of *Pan* (knuckle walker) and *Macaca* (palmigrade quadruped) found significant differences in the degree of anisotropy in the distal end of the middle phalanx [33]. This analysis compared features of trabecular bone (degree of anisotrophy "DA", elongation index "E", isotropy index "I", and BV/TV) not as a *suite* but as single, individual variables. Whereas it has been shown that the overall shape of phalanges is responsive to loads imposed during use, the internal structure of these elements has not been fully examined in this light. Increased curvature of the manual proximal phalanges is a marker of suspensory behaviors in primates [34–38]. Richmond [37] has demonstrated how curvature increases throughout ontogeny with increased use of suspensory postures, and conversely how digits of infants (typically curved as they are used to grasp onto their mothers’ fur) become increasingly flatter in quadrupedal primates as they age. Finite element analysis reveals how this curvature mitigates strain on the proximal phalanges when loads from suspensory activities are applied [37–38]. Finer discrimination between locomotor categories (quadrupedal, brachiation, quadrumanous, and knuckle walking) is revealed when the curvature values of proximal and middle phalanges of the manus (Index of Relative Curvature) are compared [39]. This locomotor signal holds true even when the indices are comprised from middle and proximal phalanges from different rays within the same manus, or with elements from different *individuals* of the same species. The Index of Relative Curvature reflects differences in how elements are loaded within a digit and across the locomotor spectrum. Knuckle walkers (*Pan* and *Gorilla*) have index values that approximate 0.85: the middle phalanges are “flatter” than the proximal and the inference can be made that these elements experience different angles of compression than the proximal phalanges while knuckle walking. In addition, when used in suspension, proximal phalanges experience tension in the palmar direction and compressive strain dorsally acting to “bend” the element [38]. Quadrupeds (*Macaca* and *Cebus*) also experience different loads at the proximal and middle phalanges. They have index values greater than 1: the middle phalanges are more curved than the proximal. In contrast, the brachiators (*Hylobates* and *Ateles*) experience similar loads upon both the middle and proximal phalanges and possess index values approximately equal to 1. Both elements are highly curved and no significant differences in curvature values occur across the manus of individual brachiators indicating relatively even distribution of weight during locomotion. Quadrumanous climbers (orangutans) have indices that (like those of knuckle walkers) approximate 0.85. However, their middle phalanges are not flattened. They simply are not as curved as the proximal. Orangutans possess significantly higher middle and proximal curvature values than the other included taxa. Like brachiators, they have long, hook-like hands which may act to mitigate the considerable amount of tensile strain that may be placed upon the digits during suspension.

Given the markedly different forces applied to the manual digits in suspension, quadrupedalism and knuckle walking, one might expect to see distinct differences in trabecular bone alignment among species engaging in these types of locomotion. As seen with the Index of Relative curvature studies, the proximal and middle phalanges may experience different loads depending on the position of the manus during movement. Primates have broad locomotor repertories. Both *Pan* and *Gorilla*, frequently travel via knuckle walking but can spend considerable amounts of time climbing and in suspension. In addition, their hands are used for a number of other tasks including grooming, tool making, and tool use. These require fine motor manipulation of the digits and may contribute to differences in manual morphology [31]. The positional behaviors of taxa included in this study (*Pan*, *Gorilla*, *Pongo*, *Hylobates*, and *Macaca*) differ in phalangeal curvature indices in a manner correlating with locomotion, and therefore it is expected that locomotor-related differences in trabecular orientation should also occur.

This paper examines the trabecular structure of the proximal and distal ends of manual middle and proximal phalanges and the metacarpal heads. The main goal is to determine if there are features of the trabecular bone that can be related to locomotor function. The middle phalanges of knuckle walkers (*Pan* and *Gorilla*) experience considerable compressive forces as they support the body during knuckle walking, with pressure focused on the 2^nd^ and 3^rd^ digits [39]. Given this, it is expected that the middle phalanges will have a dorsal-palmar alignment of the trabecular bone. In contrast, the proximal phalanges of knuckle walkers experience downward compressive strain in the knuckle-walking position, but also tensile force when the body is in a suspensory posture. These elements are expected to show a more proximodistal orientation of trabecular bone than the middle phalanges. The suspensory primates (*Pongo* and *Hylobates*) have substantial tensile and compressive forces acting on both the middle and proximal phalanges as they hang below branches and should display greater proximodistal trabecular orientation in both elements. Gibbons also experience torsional forces during fluid and fast brachiation which can be altered within a given locomotor bout by branch diameter, distance between branches, and height of branches [40–42] but it is unclear what affect this may have on trabecular structure. The quadrupeds (*Macaca*) may experience similar compressive forces on both elements if the hand is maintained in a fully palmigrade posture. It is expected that the main trabecular orientation for proximal phalanges will be palmar-dorsal and for the middle (more curved elements) proximodistal.

The metacarpal heads are loaded quite differently depending upon hand posture. Both knuckle walkers and arboreal quadrupeds can experience both tensile and compressive forces on these elements, while brachiators and quadrumanous climbers would be subjected primarily to tensile and possibly torsional forces during suspension. Previous studies [27, 30] indicate greater loading in the dorsal and palmar regions of the metacarpal heads of knuckle walkers and it is expected that the trabecular bone will be aligned along this axis. In contrast, a proximodistal alignment is expected for the suspensory and quadrupedal primates. This is the first examination of trabecular bone in manual phalanges and expands upon the previous examinations of the metacarpal head structure.

## Materials and Methods

The study examined skeletal specimens of extant apes (orangutans, gibbons, chimpanzees, and gorillas) loaned from the collections of the Harvard Museum of Comparative Zoology Cambridge, MA; and skeletal material of macaques from the Anthropology Dept. Collection of the University of Massachusetts Amherst, MA (see S1 Table for sample catalogue numbers). All the apes were wild caught, and the macaques were bred and raised in captivity. Only skeletal material from adult individuals who were free of any osteopathologies were included in the study. Taxa included in this study represent a number of locomotor categories: knuckle walkers (*Pan troglodytes* and *Gorilla gorilla*), quadrumanous climbers (*Pongo pygmaeus*), quadrupeds (*Macaca fascicularis*) and brachiators (*Hylobates lar*) (Table 1).

**Table 1:** Included samples.

Taxa	Males	Females	Locomotor category
Pan troglodytes	4	4	Knuckle walking
Gorilla gorilla	5	5	Knuckle walking
Hylobates lar	5	2	Brachiator/Suspensory
Pongo pygmaeus	1	4	Quadrumanus/suspensory
Macaca fascicularis	5		Quadruped

The 3^rd^ proximal phalanges, 3^rd^ middle phalanges, and 3^rd^ metacarpals were scanned using the HMXST Micro-CT imaging system, Harvard University. The third manual digit was chosen as it acts as the midline axis of the hand in all taxa and receives relatively greater pressure than the other digits during knuckle walking [39]. The phalanges were scanned at 70kv 80ua (apes) and 50kv 80ua (macaques).

Images were reconstructed with CT Pro software and exported as TIFF files from the VG Studiomax program. Trabecular “cubes” were extracted using Irfanview and analyzed with the SVD (star-volume distribution method in Quant 3D; [19]). Every "cube" was oriented so that the dorsal aspect of the element was at the top of the image. Because volume of interest (VOI) size and location can affect trabecular variables [43–44], the maximum amount of trabecular bone in the distal and proximal locations of the phalanges and metacarpal heads was examined. The maximum VOI was limited by the number of image slices that can be obtained for the proximodistal depth without encompassing cortical bone at the articular surfaces and empty space in the shaft. This area encompassed a large portion of trabecular bone in all selected areas for all taxa except for the middle phalanges of some male gorillas; these elements are very wide and flat, so 3 smaller trabecular cubes could be extracted. No significant differences in trabecular features were seen in the lateral, medial, and centered cube so values for the centered cube were selected for analysis. A "best fit sphere VOI" for each cube was selected using Quant 3D and the orientation parameters set to a uniform setting with 513 orientations, random rotation, and dense vectors as recommended by Ketcham and Ryan [12]. The "uniform" method creates a regular grid and the "random rotation" option helps to mitigate the bias that would come from following the pixel plans exactly during analysis [12].

**Fig 1:**
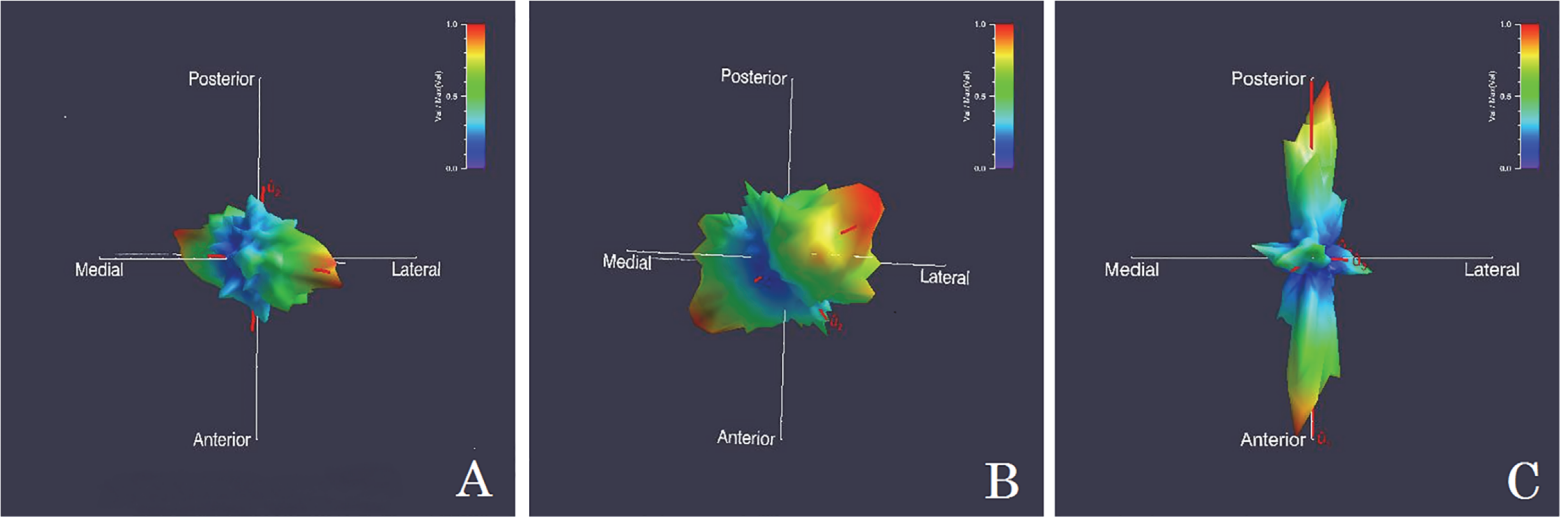
SVD Rose diagrams displaying the trabecular orientation for the proximal end of the middle phalanx. A) Knuckle Walker (female gorilla) and B) Suspensory Primate (female orangutan) main direction of trabecular orientation is towards reader (red area) in a proximo-distal alignment. Shape is rod-like for both A & B. C) Quadruped (male macaque) has a palmar-dorsal orientation and plate-like shape. (Posterior-anterior = palmar-dorsal; proximal-distal runs towards-away from viewer)

Quant 3D generated the following SVD variables: DA (degree of anisotropy; high DA values = higher degree of alignment of trabeculae), I (isotropy index; inverse of DA), E (elongation index; high E values = high elongation of trabeculae), and BV/TV (volume of trabecular bone to total volume) for the proximal and middle ends of the phalanges and the metacarpal head (S1–S5 Tables). DA, I, and E are calculated from three eigenvalues (ev_1–3_) generated by Quant 3D: DA = ev_1_ / ev_3_; I = ev_3_ / ev_1_; E = 1 – (ev_1_ / ev_3_)). The program also produces three eigenvalues (ev_1–3_) which correspond to trabecular fabric shape (ev_1_ ≈ ev_2_ ≈ ev_3_ = spherical; ev_1_ ≈ ev_2_ > ev_3_ = disc-like; ev_1_ > ev_2_ ≈ ev_3_ = rod-like) and three eigenvectors which determine trabecular orientation (each eigenvector has corresponding x, y, and z coordinates). The main direction of orientation was determined for each location based on the first SVD eigenvector values. Trabecular orientation and fabric shape were numerically coded for each location (*orientation*: 1 = medial-lateral or towards digits 2 and 4; 2 = palmar-dorsal; 3 = proximodistal; *fabric shape*: 1 = spherical; 2 = disc-like; 3 = rod-like). For trabecular orientation the “main” direction of alignment was chosen (i.e. in some cases trabecular bone was aligned in an angular direction along a proximodistal and palmar-dorsal direction and in these cases the “stronger” directionality was chosen and coded) (see Fig. 1 for SVD rose diagram depiction of shape and orientation).

Orientation, fabric shape, DA, I, E, and BV/TV were compared between sexes for each genus using t-tests in SPSS v20. These variables were also examined across taxa using the Mann-Whitney U test. Trabecular orientation and shape for each anatomical location were compared across locomotor categories using chi-square analyses. Paired t-tests were also used to compare orientation and shape of the proximal to the distal locations of the phalanges for each taxon to assess “within element” structural differences.

To assess the discriminating power of a single area of bone, a particular element, and a combination of manual locations, several Discriminant Function Analyses (DFA) were used. All DFAs compared locomotor categories for the following variables: DA, I, E, BV/TV, orientation, and shape. Five DFAs were run comparing variables of each anatomical location (i.e. all the variables of the proximal end of the proximal phalanges were included in one DFA). Two DFAs included variables of a single element: one for the proximal and distal ends of the proximal phalanges and one for the proximal and distal ends of the middle phalanges. Finally, DFA was used to compare all variables at all locations across taxa.

## Results

Orientation results are presented in Table 2, and compared with expected orientations in Table 3. Orangutans are characterized by more proximodistal alignment in all locations. Macaques have more palmar-dorsal alignment in the proximal ends of the phalanges and more proximodistal alignment in the distal ends and metacarpal heads. In contrast, knuckle walkers and gibbons have more proximodistal alignment in the proximal ends of the phalanges and more palmar-dorsal in the distal ends. Knuckle walkers and gibbons differ in the orientation of the metacarpal trabecular bone: knuckle walkers are more palmar-dorsally aligned and gibbons more proximodistally aligned.

**Table 2:** Percentage of individuals for trabecular orientation in examined regions by genus.

Genus	Digit and Location				
	Middle Phalanx		Proximal Phalanx		Metacarpal Head
	Proximal	Distal	Proximal	Distal	
*Pan*	**75% prox-dis**	12% prox-dis	**75% prox-dis**	25% prox-dis	17% prox-dis
	12.5% palm-dor	**88% palm-dor**	12.5% palm-dor	**50% palm-dor**	**66% palm-dor**
	12.5% med-lat		12.5% med-lat	25% med-lat	17% med-lat
*Gorilla*	**100% prox-dis**	30% prox-dis	**100% prox-dis**	**80% palm-dor**	22% prox-dis
		**70% palm-dor**		20% med-lat	**78% palm-dor**
*Pongo*	**100% prox-dis**	**80% prox-dis**	**100% prox-dis**	**80% prox-dis**	**100% prox-dis**
		20% palm-dor		20% med-lat	
*Hylobates*	**86% prox-dis**	14% prox-dis	**72% prox-dis**	14% prox-dis	**100% prox-dis**
	14% palm-dor	**72% palm-dor**	28% palm-dor	**72% palm-dor**	
		14% med-lat		14% med-lat	
*Macaca*	33% prox-dis	**83% prox-dis**	**80% palm-dor**	**40% prox-dis**	**60% prox-dis**
	**67% palm-dor**	17% palm-dor	20% med-lat	**40% palm-dor**	40% palm-dor
				20% med-lat	

(Greatest percentage in bold.)

prox-dis = proximodistal orientation; palm-dor = palmar-dorsal orientation; med-lat = medial lateral orientation.

**Table 3:** Hypothesized (expected) and observed primary trabecular orientation for taxa.

Locomotor Category	Included Taxa	Expected Trabecular Orientation	Primary Observed Orientation
Knuckle walkers	*Pan* & *Gorilla*	MD: palmar-dorsal	MDprox: proximodistal
			MDdis: palmar-dorsal
		PX: proximodistal	PXprox: proximodistal
			PXdis: palmar-dorsal
		MC: palmar-dorsal	MC: palmar-dorsal
Quadrupeds	*Macaca*	MD: proximodistal	MDprox: palmar-dorsal
			MDdis: proximodistal
		PX: palmar-dorsal	PXprox: palmar-dorsal
			PXdis: proximodistal & palmar-dorsal
		MC: palmar-dorsal	MC: proximodistal
Suspensory	*Hylobates* & *Pongo*	MD: proximodistal	MDprox: proximodistal
			MDdis: proximodistal & palmar-dorsal
		PX: palmar-dorsal	PXprox: proximodistal
			PXdis: proximodistal & palmar-dorsal
		MC: palmar-dorsal	MC: proximodistal

Digit- MD = middle phalanx; PX = proximal phalanx; MC = metacarpal.

Location- “dis” = distal end; “prox” = proximal end

### Differences between the sexes

T-tests reveal no significant differences in the trabecular orientation and trabecular fabric shape between the sexes at any location (proximal and distal ends of the phalanges and the metacarpal head) for any taxon. There were also no differences between the sexes for DA, I, E and BV/TV in the gibbon or orangutan samples. Significant differences were seen between male and female chimpanzees in the trabecular variables of the middle phalanges: males have a greater degree of anisotropy in the proximal (t = 3.93, df = 6; P = 0.01) and distal ends (t = 3.13, df = 6, P = 0.04) of the middle phalanges and females have significantly greater I values at both locations (proximal end t = -3.5, df = 6, P = 0.03; distal end t = -3.2, df = 6, P = 0.02). Female chimpanzees also show increased BV/TV in the proximal ends of the middle phalanges (t = -5.5, df = 6, P = 0.008) and proximal phalanges (t = -5.8, df = 6, P = 0.001). Gorilla males and females differ only in BV/TV for the proximal ends of the middle and proximal phalanges: females have increased BV/TV at both locations (middle phalanges t = -2.5, df = 8, P = 0.046; proximal phalanges t = -2.96, df = 8, P = 0.018). Subsequent analyses were conducted on pooled male and female samples.

### Differences across taxa and locomotor categories

Taken alone, variables (DA, I, E, and BV/TV) show no discernable pattern of differences across taxa that can be related to function or phylogenetic relationships (Table 4). However orientation does show significant differences among taxa using different methods of locomotion in several locations. Chimpanzees and gorillas differ significantly from orangutans, and gibbons in trabecular bone orientation of the metacarpal head. Macaques differ significantly in trabecular orientation from the apes in regions of the phalanges. Orientation does not differ between primates attributed to the same locomotor category in any examined region.

**Table 4:** Mann-Whitney U test results for taxa comparisons.

Genus Comparisons	Digit and Location				
	Middle Phalanx		Proximal Phalanx		Metacarpal Head
	Proximal	Distal	Proximal	Distal	
*Pan—Gorilla*		BV/TV (0.03)	DA (0.02)		DA (0.01)
			I (0.02)		I (0.01)
			BV/TV (0.03)		
*Pan—Pongo*		Orientation (0.045)	BV/TV (0.01)		Orientation (0.02)
*Pan—Hylobates*		BV/TV (0.01)			Orientation (0.015)
*Pan—Macaca*	Shape (0.02)	Orientation (0.045)	BV/TV (0.01)		BV/TV (0.004)
			Orientation (0.045)		
*Gorilla—Pongo*	E (0.01)				DA (0.01)
	BV/TV (0.04)				I (0.01)
					Orientation (0.019)
*Gorilla—Hylobates*			DA (0.01)		DA (0.05)
			I (0.01)		
			Orientation (0.01)		Orientation (0.012)
*Gorilla—Macaca*	Orientation (0.013)		E (0.03)		DA (0.03)
			BV/TV (0.01)		I (0.03)
			Orientation (0.001)		BV/TV (0.02)
*Pongo—Hylobates*	E (0.03)			BV/TV (0.05)	
*Pongo—Macaca*	E (0.02)		Orientation (0.008)		DA (0.01)
	Shape (0.03)				I (0.01)
	Orientation (0.03)				BV/TV (0.01)
*Hylobates—Macaca*	Shape (0.05)	BV/TV (0.05)	DA (0.03)		BV/TV (0.004)
			I (0.03)		
			BV/TV (0.01)		
		Orientation (0.048)	Orientation (0.03)		

Only variables with significant differences presented. Significance values in parentheses.

A similar result is seen when the taxa are lumped into locomotor categories (Table 5). As there were no significant differences in DA, I, trabecular orientation or shape for orangutans (quadrumanous climbers) and gibbons (brachiators), they were both included in a broader “suspensory” category for this comparison. Quadrupeds differ from suspensory primates in the orientation of trabecular bone in the proximal ends of both phalanges. Knuckle walkers show significant differences with quadrupeds in trabecular orientation at the distal end of the middle phalanges and proximal end of the proximal phalanges, and they are significantly different from suspensory primates in trabecular orientation of the metacarpal head.

**Table 5:** Mann-Whitney U test results for locomotor category comparisons. Significance values in parenthesis.

Locomotor Comparisons	Digit and Location				
	Middle Phalanx		Proximal Phalanx		Metacarpal Head
	Proximal	Distal	Proximal	Distal	
Knuckle walkers—Suspensory Primates	BV/TV (0.04)		BV/TV (0.05)		E (0.03)
					Orientation (0.001)
Knuckle walkers—Quadrupeds		E (0.03)	E (0.01)	DA (0.02)	BV/TV (0.01)
			BV/TV (0.001)	I (0.02)	
		Orientation (0.027)	Orientation (0.002)		
Suspensory Primates—Quadrupeds	Shape (0.04)		Shape (0.04)		BV/TV (0.002)
			BV/TV (0.01)		
	Orientation (0.05)		Orientation (0.002)		

#### Trabecular bone orientation

**Fig 2:**
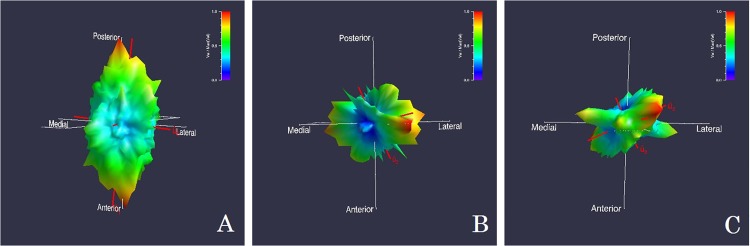
SVD Rose diagrams displaying the trabecular orientation for the metacarpal head. A) Knuckle Walker (female chimpanzee) has a palmar-dorsal trabecular orientation and plate-like shape. B) Suspensory Primate (male gibbon) and C) Quadruped (male macaque) have a proximal-dorsal orientation and are approximately rod-like in shape.

Chi-square analyses comparing trabecular orientation between the locomotor categories revealed significant differences in several locations. Trabecular orientation was significantly different in the proximal end of the middle phalanges (X^2^ = 0.009), the proximal end of the proximal phalanges (X^2^ = 0.001), and the metacarpal head (X^2^ = 0.002). In the proximal end of the middle and proximal phalanges, knuckle walkers and suspensory primates have a greater proportion of individuals with a proximodistal alignment (knuckle walkers—89% in both locations; Suspensory—92% of middle and 83% of proximal phalanges). Quadrupeds have a greater proportion of palmar-dorsal alignments in both areas (67% in both locations) (Fig. 1). In contrast to the structure of the phalanges, knuckle walkers have a greater proportion of palmar-dorsal orientation (73%) in the metacarpal head. Suspensory primates have 100% of individuals with a proximodistal alignment in this area, and quadrupeds display nearly equal amounts of proximodistal (60%) and palmar-dorsal (40%) orientations (Fig. 2).

#### Trabecular bone shape

These same areas (proximal ends of the middle and proximal phalanges and metacarpal head) also show significant differences in trabecular bone shape. In the proximal end of the middle phalanges (X^2^ = 0.028), knuckle walkers have a greater proportion of individuals with “rod-like” trabeculae (72%) and a lesser number with more disc-like trabeculae (28%). Suspensory primates also have a greater proportion of individuals with rod-like trabeculae (93%) and quadrupeds have an equal percentage of “spherical”, rod-like, and disc-like trabeculae. A similar pattern is seen in the proximal end of the proximal phalanges (X^2^ = 0.019) for the knuckle walkers and suspensory primates: both have a greater proportion of individuals with rod-like trabeculae (knuckle walkers = 72%; suspensory = 83%). Quadrupeds have 83% of individuals with disc-like trabeculae. Differences in the metacarpal head approach significance (X^2^ = 0.059). In this region knuckle walkers and quadrupeds have disc-like trabeculae (knuckle walkers = 73%; quadrupeds = 100%), and suspensory primates show nearly even proportions of “spherical” (31%), rod-like (38%), and disc-like (31%) trabeculae.

#### "Within" element differences

Paired t-tests revealed significant differences between the proximal and distal ends of the phalanges for two taxa. Gorillas have significantly different trabecular orientation at the proximal and distal ends of both phalanges (middle phalanges: t = 4.6, df = 9, P < 0.001; proximal phalanges: t = 9, df = 9, P < 0.001). Gibbons showed significant differences in both shape and orientation. They differ significantly in orientation between the proximal and distal ends of both phalanges (middle phalanges: t = 3.3, df = 6, P = 0.017; proximal phalanges: t = 3.9, df = 6, P = 0.008) and in shape in the proximal and distal ends of the proximal phalanges (t = 6, df = 6, P = 0.001). Both gorillas and gibbons are characterized by a proximodistal alignment at the proximal ends of the phalanges and a more palmar-dorsal alignment at the distal ends.

### Discriminant Function Analyses

#### Middle phalanges

The Discriminant function analyses for specific locations were successful at discriminating between locomotor categories. The functions for the *proximal* end of the middle phalanges were significant (X^2^ = 30.5, df = 12, P = 0.002). This DFA successfully classified 74.3% of original grouped cases. Function 1 accounts for 81% of the variance and polarizes macaques with lower orientation and shape values (more palmar-dorsal, medial-lateral orientation and more spherical and disc-shaped trabeculae) from knuckle walkers and suspensory primates (more proximodistal orientation). Function 2 polarizes knuckle walkers who have higher DA, and less BV/TV and suspensory primates. The functions for the DFA of the *distal* end of the middle phalanges were not significant. However this analysis correctly classified 71.4% of the original groups. Quadrupeds and suspensory primates with higher E and orientation values are distinguished from knuckle walkers along function 1 (which accounts for 51.8% of variance). Function 2 (48.2% of the variance) differentiates between the three groups with quadrupeds having the highest BV/TV and orientation values and suspensory primates (with the lowest values for BV/TV and orientation). Knuckle walkers fall in the middle. The DFA using variables for both the *proximal* and *distal* ends of the middle phalanges was successful at classifying 85.7% of original grouped cases. The functions were highly significant (X^2^ = 42.2, df = 24, P = 0.012) and functions 1 and 2 accounted for 62.9% and 37.1% of the variance respectively. All three categories are separated along Function 1 with suspensory primates having the greatest *proximal* end shape and orientation values (more rod-like and proximodistal alignment), knuckle walkers in the middle and quadrupeds the lowest values. Function 2 separates the knuckle walkers with more proximodistal alignment of the *distal* region and less BV/TV from both the quadrupeds and suspensory primates.

#### Proximal phalanges

The DFA functions for the *proximal* end of the proximal phalanges are highly significant (X^2^ = 56.3, df = 12, P < 0.001) and correctly classified 82.9% of the originally grouped cases. Function 1 accounts for 81.9% of the variance and separates the quadrupeds with lower BV/TV and orientation values (more palmar-dorsal alignment) from the knuckle walkers and suspensory primates (proximodistal alignment). Function 2 separates the suspensory primates with higher I values and lower DA from both knuckle walkers and quadrupeds. The DFA functions for the *distal* end of the proximal phalanges were not significant. The DFA correctly classified 62.9% of original groups. Most of the variance is accounted for by function 1 (87.3%) and delineates between all three groups: knuckle walkers have greater I, BV/TV, and orientation values (more proximo-distal alignment) than quadrupeds with the lowest and suspensory primates in the middle. Function 2 weakly separates knuckle walkers and quadrupeds (with higher DA) from suspensory primates. The classification success increases to 85.7% when the DFA is run with variables from both the *proximal* and *distal* ends. The functions were highly significant (X^2^ = 58.4, df = 24, P < 0.001), and most of the variance is accounted for by function 1 (80.7%). This function delineates between all three groups with knuckle walkers having the greatest BV/TV and orientation values (more proximo-distal alignment) in the *proximal* end, macaques the lowest and suspensory primates in the middle. Function 2 separates out the suspensory primates with higher *proximal* end I values and lower *proximal* end DA from the knuckle walkers and quadrupeds.

#### Metacarpal heads

The DFA for the metacarpal head variables correctly classified 93.5% of the original grouped cases. The functions were highly significant (X^2^ = 53.9, df = 12, P < 0.001), and Function 1 accounts for 70.2% of the variance and function 2 for 29.8%. Function 1 differentiates between all three categories with knuckle walkers having the highest BV/TV and lowest orientation values (more palmar-dorsal alignment), quadrupeds the lowest BV/TV and higher orientation values and suspensory primates in the middle (both have more proximodistal alignment). Function 2 separates the suspensory primates from the knuckle walkers and quadrupeds. The suspensory primates have higher E and shape values (more rod- and disc-like), and higher orientation values (proximodistal alignment).

#### All locations

**Fig 3:**
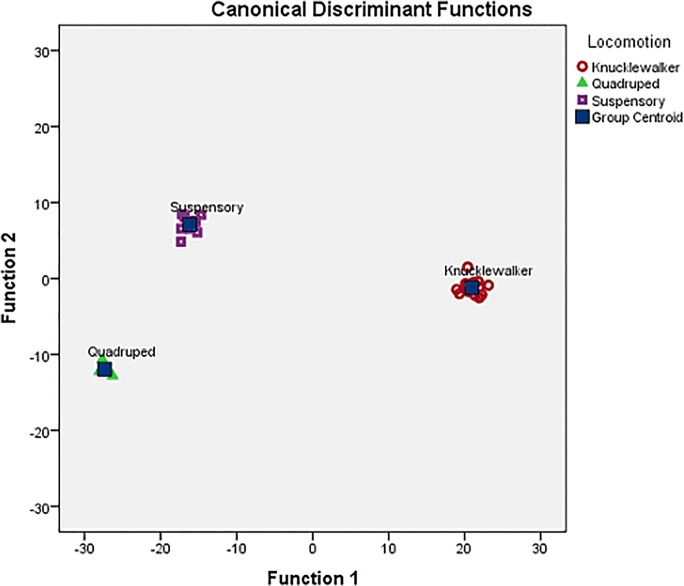
Discriminant Function Plot for all included regions (proximal and distal ends of the middle and proximal phalanges and the metacarpal head). Along Function 1, knuckle walkers have high BV/TV in the proximal and distal ends of the proximal phalanges and proximal end of the middle phalanges and more palmar-dorsal alignment in the metacarpal head and distal end of the proximal phalanges. Quadrupeds and suspensory primates have more proximo-distal alignment in those regions.

The last DFA was run with the variables (DA, I, E, BV/TV, shape and orientation) for all 5 locations (proximal and distal ends of both phalanges and the metacarpal head). The functions were highly significant (X^2^ = 145.1, df = 56, P > 0.001) and 100% of original cases were correctly classified. DFA cross validation only shows a correct classification of 48.5% of cases. Function 1 accounts for 91.1% of the variance and delineates between all three locomotor categories: knuckle walkers are characterized by high BV/TV in the proximal and distal ends of the proximal phalanges and proximal end of the middle phalanges and lower orientation values in the metacarpal head and distal end of the proximal phalanges (more palmar-dorsal alignment). Quadrupeds have the lowest BV/TV values and higher orientation values in the aforementioned areas, and suspensory primates fall in between quadrupeds and knuckle walkers (closer to the quadrupeds) (Fig. 3). Function 2 differentiates between suspensory primates and the other locomotor categories. Suspensory primates have the higher metacarpal shape and orientation values (“rod-like” in a proximo-distal alignment) and higher orientation values for the proximal end of the middle phalanges.

When the analysis is run with I and E removed to eliminate some variable redundancy (I = inverse of DA, E = 1- DA) the functions remain highly significant (X2 = 82.7, df = 40, P < 0.001), 100% of the original cases are correctly classified, and cross validation increases to 64.5% correct classification. The discriminatory factors remain the same on functions 1 and 2 as in the above analysis (function 1–82.4% of variance; function 2–17.6%).

## Discussion

Trabecular orientation within the manus is sensitive to locomotor function. Taxa assigned to the same locomotor categories have similar trabecular orientation. The latter can be related to the stresses placed on the digits during locomotion. Macaques show a pattern of curvature within the phalanges that somewhat fits the predicted expectation of greater proximodistal alignment in the curved middle phalanges and more palmar-dorsal alignment in the “flatter” proximal phalanges. They do possess increased proximodistal alignment in the distal end of the middle phalanges but all other areas show a greater percentage of palmar-dorsal alignment.

The highly suspensory orangutans, as predicted, had more proximodistal alignment of the trabecular bone at all locations examined. In addition to the extremely high curvature values of these animals, a more proximodistal alignment of the trabecular bone may help to buffer against tensile forces during suspension. The gibbons, although not significantly different from orangutans in orientation at any location, do show some differences from them in distal ends of the phalanges. It was predicted that these agile, brachiators would also have a more proximodistal alignment of trabecular bone in all examined areas to also mitigate against tensile strain. In the proximal ends of the phalanges and in the metacarpal head, this prediction holds true, but in the distal ends of the proximal and middle phalanges, there is a greater percentage of individuals with a more palmar-dorsal alignment. Richmond [38] has demonstrated using FEA that there is compressive strain dorsally on the proximal phalanges when loaded as in suspension. It is possible that more compressive forces are acting on these areas due to gravitational pull during suspension.

Knuckle walkers show a similar pattern to gibbons in the orientation of trabecular bone in the phalanges. It was predicted that palmar-dorsal alignment would be present in both the proximal and distal ends of the middle phalanges due to the high compressive forces that knuckle walking places upon these elements. However, like the gibbons, knuckle walkers have a more palmar-dorsal alignment in the distal ends of both phalanges and a more proximodistal alignment in the proximal ends. Pressure outputs show increased pressure application in the proximal and distal regions of the middle phalanges [39] so it is unclear why a more proximodistal alignment is shown in the proximal end of this element. Strain gage analyses of the phalanges have not been conducted so it can not be determined for sure what level and direction of strain is being placed upon the phalanges during knuckle walking and suspension. It may be that the proximal ends of the phalanges are experiencing increased tensile forces during suspension. Both chimpanzees and gorillas have a palmar-dorsal orientation of trabecular bone in the metacarpal head. Results obtained for this region coincide with earlier studies [27] that show more activity in the palmar and dorsal regions of the metacarpal head. This orientation could mitigate strain caused via hyperextension at the metacarpophalangeal joint during knuckle walking.

Although several of the trabecular variables (DA, I, E, BV/TV) taken alone show no pattern that can be related to locomotor function, orientation and shape have proven to differentiate successfully at several locations in the manus. Shape and orientation of the *metacarpal heads* and the *proximal ends* of the phalanges discriminate between locomotor categories. Knuckle walkers and suspensory primates are characterized by having greater proximodistal alignment and rod-like trabeculae in the proximal ends of the phalanges, whereas quadrupeds have a more palmar-dorsal alignment and a variety of trabecular form. The metacarpal head shows distinct differences between the groups: knuckle walkers have a palmar-dorsal alignment and disc-like shape, suspensory taxa have a proximodistal alignment and rod-like shape and quadrupeds have a proximodistal alignment and disc-like shape. Again, these two variables, taken alone are successful differentiating between primates practicing different methods of locomotion.

The additional variables (DA, I, E, and BV/TV) discriminate between taxa if coupled with shape and orientation. Using DFA, each location within the manus had greater than 60% accuracy in classifying individuals into three locomotor groups (knuckle walking, quadrupedal, or suspensory). Accuracy increased when regions of the same element were examined together, and reached 100% when variables of all five regions were examined. This is an extremely attractive tool to discern possible locomotor functions within extinct primates as it can be used on a fragmentary element.

## Conclusions

Trabecular orientation and fabric shape show distinct differences between primates practicing different modes of locomotion in 3 areas of the manus (metacarpal head and proximal ends of both the proximal and middle phalanges). In addition, when a *suite* of trabecular variables (DA, I, E, BV/TV, shape and orientation) are considered as a unit, the correct classification of a primate into their locomotor category is successful at all examined zones. Knuckle walkers have a distinct pattern of alignment and trabecular fabric shape in the metacarpal head that distinguish them from suspensory and quadrupedal primates. Being able to use isolated zones to predict locomotor propensity is an attractive tool given the fragmentary nature of the fossil record. These manual differences in trabecular structure can be related to locomotor use. Micro CT scans of the manus can help to determine patterns of locomotion in extinct hominoids in a non-destructive manner and may provide insight into the origins of knuckle walking in the hominin lineage.

## Supporting Information

S1 Table Star Volume Distribution (SVD) variables for the Proximal End of the Middle Phalanx (Digit 3). DA- Degree of Anistrophy; I- Isotropy Index; E- Elongation Index; BV/TV- Bone Volume. (Shape Code: 1 = spherical; 2 = disc-like; 3 = rod-like; Orientation Code: 1 = medial-lateral or towards digits 2 and 4; 2 = palmar-dorsal; 3 = proximodistal). ID Numbers: MCZ = Museum of Comparative Zoology (Harvard University); APC = Anthropological Primate Collection (University of Massachusetts, Amherst)
(DOCX)

S2 Table Star Volume Distribution (SVD) variables for the Distal End of the Middle Phalanx (Digit 3). See legend of S1 Table for code definitions.
(DOCX)

S3 Table Star Volume Distribution (SVD) variables for the Proximal End of the Proximal Phalanx (Digit 3). See legend of S1 Table for code definitions.
(DOCX)

S4 Table Star Volume Distribution (SVD) variables for the Distal End of the Proximal Phalanx (Digit 3). See legend of S1 Table for code definitions.
(DOCX)

S5 Table Star Volume Distribution (SVD) variables for the Metacarpal Heads (Digit 3). See legend of S1 Table for code definitions.
(DOCX)
