# Supplementary material for: Trabecular Architecture of the Manual Elements Reflects Locomotor Patterns in Primates
Source: PLoS One. 2015 Mar 20;10(3):e0120436. doi: 10.1371/journal.pone.0120436 (PMC4368714; doi:10.1371/journal.pone.0120436)
Supplement: S1 Table — DA- Degree of Anistrophy; I- Isotropy Index; E- Elongation Index; BV/TV- Bone Volume. (Shape Code: 1 = spherical; 2 = disc-like; 3 = rod-like; Orientation Code: 1 = medial-lateral or towards digits 2 and 4; 2 = palmar-dorsal; 3 = proximodistal). ID Numbers: MCZ = Museum of Comparative Zoology (Harvard University); APC = Anthropological Primate Collection (University of Massachusetts, Amherst) (DOCX) [file pone.0120436.s001.docx]

Supporting Information

Table S1. Star Volume Distribution (SVD) variables for the Proximal End of the Middle Phalanx (Digit 3)

| **ID NUMBER** | **SEX** | **GENUS** | **SVD DA** | **SVD I** | **SVD E** | **SVD BV/TV** | **Shape Code** | **Orientation Code** |
| --- | --- | --- | --- | --- | --- | --- | --- | --- |
| MCZ19187 | M | *Pan Troglodytes* | 3.4 | 0.29 | 0.56 | 0.34 | 3 | 3 |
| MCZ20041 | M | *Pan Troglodytes* | 3.89 | 0.26 | 0.67 | 0.33 | 3 | 3 |
| MCZ23163 | M | *Pan Troglodytes* | 3.88 | 0.26 | 0.63 | 0.35 | 3 | 3 |
| MCZ48686 | M | *Pan Troglodytes* | 4.66 | 0.21 | 0.71 | 0.31 | 3 | 3 |
| MCZ26847 | F | *Pan Troglodytes* | 3.27 | 0.31 | 0.47 | 0.48 | 3 | 3 |
| MCZ26849 | F | *Pan Troglodytes* | 1.58 | 0.63 | 0.23 | 0.56 | 3 | 1 |
| MCZ15312 | F | *Pan Troglodytes* | 1.81 | 0.55 | 0.29 | 0.44 | 3 | 3 |
| APC232 | F | *Pan Troglodytes* | 1.82 | 0.55 | 0.33 | 0.57 | 3 | 2 |
| APC77 | M | *Macaca mulatta* | 4.44 | 0.23 | 0.35 | 0.16 | 2 | 2 |
| APC88 | M | *Macaca mulatta* | 1.2 | 0.83 | 0.13 | 0.4 | 1 | 2 |
| APC224 | M | *Macaca mulatta* | 4.6 | 0.22 | 0.36 | 0.28 | 2 | 2 |
| APC279 | M | *Macaca mulatta* | 1.13 | 0.89 | 0.08 | 0.5 | 1 | 2 |
| APC286 | M | *Macaca mulatta* | 2.82 | 0.36 | 0.56 | 0.33 | 3 | 3 |
| MCZ20039 | M | *Gorilla gorilla* | 3.34 | 0.3 | 0.25 | 0.23 | 2 | 3 |
| MCZ29048 | M | *Gorilla gorilla* | 2.67 | 0.37 | 0.37 | 0.42 | 2 | 3 |
| MCZ29049 | M | *Gorilla gorilla* | 2.99 | 0.33 | 0.44 | 0.37 | 3 | 3 |
| MCZ23160 | M | *Gorilla gorilla* | 3.25 | 0.31 | 0.35 | 0.38 | 3 | 3 |
| MCZ23162 | M | *Gorilla gorilla* | 2.78 | 0.36 | 0.54 | 0.37 | 3 | 3 |
| MCZ17684 | F | *Gorilla gorilla* | 3.56 | 0.28 | 0.52 | 0.38 | 3 | 3 |
| MCZ37264 | F | *Gorilla gorilla* | 5.22 | 0.19 | 0.48 | 0.49 | 2 | 3 |
| MCZ38326 | F | *Gorilla gorilla* | 3.3 | 0.3 | 0.34 | 0.45 | 2 | 3 |
| MCZ29047 | F | *Gorilla gorilla* | 2.9 | 0.35 | 0.57 | 0.46 | 3 | 3 |
| MCZ26850 | F | *Gorilla gorilla* | 2.3 | 0.43 | 0.32 | 0.45 | 2 | 3 |
| MCZ37362 | M | *Pongo pygmaeus* | 3.59 | 0.28 | 0.46 | 0.27 | 3 | 3 |
| MCZ37365 | F | *Pongo pygmaeus* | 4.19 | 0.24 | 0.59 | 0.39 | 3 | 3 |
| MCZ37363 | F | *Pongo pygmaeus* | 5.29 | 0.19 | 0.64 | 0.28 | 3 | 3 |
| MCZ50958 | F | *Pongo pygmaeus* | 6.12 | 0.16 | 0.69 | 0.19 | 3 | 3 |
| MCZ50960 | F | *Pongo pygmaeus* | 2.65 | 0.38 | 0.6 | 0.36 | 3 | 3 |
| MCZ41541 | M | *Hylobates lar* | 2.01 | 0.5 | 0.21 | 0.37 | 2 | 2 |
| MCZ41529 | M | *Hylobates lar* | 2.72 | 0.37 | 0.51 | 0.33 | 3 | 3 |
| MCZ41534 | M | *Hylobates lar* | 3.83 | 0.26 | 0.61 | 0.44 | 3 | 3 |
| MCZ41532 | M | *Hylobates lar* | 4.02 | 0.25 | 0.39 | 0.37 | 3 | 3 |
| MCZ41531 | M | *Hylobates lar* | 2.18 | 0.46 | 0.3 | 0.22 | 3 | 3 |
| MCZ41540 | F | *Hylobates lar* | 4.86 | 0.21 | 0.45 | 0.33 | 3 | 3 |
| MCZ41543 | F | *Hylobates lar* | 2.65 | 0.38 | 0.4 | 0.51 | 3 | 3 |

DA- Degree of Anistrophy; I- Isotropy Index; E- Elongation Index; BV/TV- Bone Volume. (Shape Code: 1 = spherical; 2 = disc-like; 3 = rod-like; Orientation Code: 1 = medial-lateral or towards digits 2 and 4; 2 = palmar-dorsal; 3 = proximodistal). ID Numbers: MCZ = Museum of Comparative Zoology (Harvard University); APC= Anthropological Primate Collection (University of Massachusetts, Amherst)
