# Supplementary material for: Trabecular Architecture of the Manual Elements Reflects Locomotor Patterns in Primates
Source: PLoS One. 2015 Mar 20;10(3):e0120436. doi: 10.1371/journal.pone.0120436 (PMC4368714; doi:10.1371/journal.pone.0120436)
Supplement: S3 Table — See legend of S1 Table for code definitions. (DOCX) [file pone.0120436.s003.docx]

Table S3. Star Volume Distribution (SVD) variables for the Proximal End of the Proximal Phalanx (Digit 3)

| **ID NUMBER** | **SEX** | **GENUS** | **SVD DA** | **SVD I** | **SVD E** | **SVD BV/TV** | **Shape Code** | **Orientation Code** |
| --- | --- | --- | --- | --- | --- | --- | --- | --- |
| MCZ19187 | M | *Pan Troglodytes* | 2.54 | 0.39 | 0.47 | 0.39 | 3 | 3 |
| MCZ20041 | M | *Pan Troglodytes* | 3.15 | 0.32 | 0.57 | 0.36 | 3 | 3 |
| MCZ23163 | M | *Pan Troglodytes* | 3.77 | 0.27 | 0.58 | 0.34 | 3 | 3 |
| MCZ48686 | M | *Pan Troglodytes* | 4.54 | 0.22 | 0.75 | 0.32 | 3 | 3 |
| MCZ26847 | F | *Pan Troglodytes* | 1.65 | 0.6 | 0.18 | 0.52 | 1 | 2 |
| MCZ26849 | F | *Pan Troglodytes* | 2.07 | 0.48 | 0.26 | 0.44 | 2 | 3 |
| MCZ15312 | F | *Pan Troglodytes* | 2.24 | 0.45 | 0.34 | 0.48 | 3 | 3 |
| APC232 | F | *Pan Troglodytes* | 4.56 | 0.22 | 0.34 | 0.51 | 3 | 1 |
| APC77 | M | *Macaca mulatta* | 4.83 | 0.21 | 0.16 | 0.18 | 2 | 1 |
| APC88 | M | *Macaca mulatta* | 2.82 | 0.36 | 0.33 | 0.35 | 2 | 2 |
| APC224 | M | *Macaca mulatta* | 10.11 | 0.1 | 0.13 | 0.14 | 2 | 2 |
| APC279 | M | *Macaca mulatta* | 3.28 | 0.3 | 0.54 | 0.18 | 3 | 2 |
| APC286 | M | *Macaca mulatta* | 4.01 | 0.25 | 0.18 | 0.21 | 2 | 2 |
| MCZ20039 | M | *Gorilla gorilla* | 3.68 | 0.27 | 0.5 | 0.26 | 3 | 3 |
| MCZ29048 | M | *Gorilla gorilla* | 4.51 | 0.22 | 0.62 | 0.27 | 3 | 3 |
| MCZ29049 | M | *Gorilla gorilla* | 3.84 | 0.26 | 0.26 | 0.31 | 2 | 3 |
| MCZ23160 | M | *Gorilla gorilla* | 10.2 | 0.1 | 0.36 | 0.36 | 2 | 3 |
| MCZ23162 | M | *Gorilla gorilla* | 9.69 | 0.1 | 0.41 | 0.31 | 2 | 3 |
| MCZ17684 | F | *Gorilla gorilla* | 6.74 | 0.15 | 0.7 | 0.31 | 3 | 3 |
| MCZ37264 | F | *Gorilla gorilla* | 3.37 | 0.3 | 0.53 | 0.39 | 3 | 3 |
| MCZ38326 | F | *Gorilla gorilla* | 4.8 | 0.21 | 0.47 | 0.37 | 3 | 3 |
| MCZ29047 | F | *Gorilla gorilla* | 4.15 | 0.24 | 0.67 | 0.42 | 3 | 3 |
| MCZ26850 | F | *Gorilla gorilla* | 3.77 | 0.27 | 0.66 | 0.41 | 3 | 3 |
| MCZ37362 | M | *Pongo pygmaeus* | 1.23 | 0.82 | 0.11 | 0.37 | 1 | 3 |
| MCZ37365 | F | *Pongo pygmaeus* | 4.89 | 0.2 | 0.75 | 0.27 | 3 | 3 |
| MCZ37363 | F | *Pongo pygmaeus* | 4.5 | 0.22 | 0.66 | 0.3 | 3 | 3 |
| MCZ50958 | F | *Pongo pygmaeus* | 2.59 | 0.39 | 0.49 | 0.21 | 3 | 3 |
| MCZ50960 | F | *Pongo pygmaeus* | 2.35 | 0.43 | 0.55 | 0.3 | 3 | 3 |
| MCZ41541 | M | *Hylobates lar* | 2.04 | 0.49 | 0.29 | 0.36 | 3 | 2 |
| MCZ41529 | M | *Hylobates lar* | 3.95 | 0.25 | 0.53 | 0.29 | 3 | 3 |
| MCZ41534 | M | *Hylobates lar* | 2.68 | 0.37 | 0.53 | 0.38 | 3 | 3 |
| MCZ41532 | M | *Hylobates lar* | 2.52 | 0.4 | 0.54 | 0.26 | 3 | 3 |
| MCZ41531 | M | *Hylobates lar* | 2 | 0.5 | 0.37 | 0.35 | 3 | 2 |
| MCZ41540 | F | *Hylobates lar* | 3.88 | 0.26 | 0.64 | 0.36 | 3 | 3 |
| MCZ41543 | F | *Hylobates lar* | 1.86 | 0.54 | 0.09 | 0.39 | 2 | 3 |
