# Supplementary material for: Trabecular Architecture of the Manual Elements Reflects Locomotor Patterns in Primates
Source: PLoS One. 2015 Mar 20;10(3):e0120436. doi: 10.1371/journal.pone.0120436 (PMC4368714; doi:10.1371/journal.pone.0120436)
Supplement: S4 Table — See legend of S1 Table for code definitions. (DOCX) [file pone.0120436.s004.docx]

Table S4. Star Volume Distribution (SVD) variables for the Distal End of the Proximal Phalanx (Digit 3)

| **ID NUMBER** | **SEX** | **GENUS** | **SVD DA** | **SVD I** | **SVD E** | **SVD BV/TV** | **Shape Code** | **Orientation Code** |
| --- | --- | --- | --- | --- | --- | --- | --- | --- |
| MCZ19187 | M | *Pan Troglodytes* | 2.21 | 0.45 | 0.42 | 0.39 | 3 | 3 |
| MCZ20041 | M | *Pan Troglodytes* | 1.67 | 0.6 | 0.16 | 0.49 | 1 | 1 |
| MCZ23163 | M | *Pan Troglodytes* | 1.21 | 0.83 | 0.07 | 0.39 | 1 | 1 |
| MCZ48686 | M | *Pan Troglodytes* | 4.41 | 0.23 | 0.41 | 0.44 | 2 | 3 |
| MCZ26847 | F | *Pan Troglodytes* | 1.72 | 0.58 | 0.22 | 0.53 | 2 | 2 |
| MCZ26849 | F | *Pan Troglodytes* | 2.09 | 0.48 | 0.3 | 0.5 | 3 | 2 |
| MCZ15312 | F | *Pan Troglodytes* | 2.35 | 0.43 | 0.32 | 0.56 | 3 | 2 |
| APC232 | F | *Pan Troglodytes* | 5.2 | 0.19 | 0.71 | 0.39 | 3 | 2 |
| APC77 | M | *Macaca mulatta* | 11.71 | 0.09 | 0.75 | 0.27 | 3 | 1 |
| APC88 | M | *Macaca mulatta* | 2.6 | 0.38 | 0.53 | 0.49 | 3 | 3 |
| APC224 | M | *Macaca mulatta* | 1.94 | 0.51 | 0.1 | 0.39 | 2 | 3 |
| APC279 | M | *Macaca mulatta* | 2.42 | 0.41 | 0.18 | 0.32 | 2 | 2 |
| APC286 | M | *Macaca mulatta* | 6.62 | 0.15 | 0.11 | 0.48 | 2 | 2 |
| MCZ20039 | M | *Gorilla gorilla* | 2.5 | 0.4 | 0.31 | 0.58 | 2 | 1 |
| MCZ29048 | M | *Gorilla gorilla* | 2.2 | 0.45 | 0.42 | 0.42 | 3 | 2 |
| MCZ29049 | M | *Gorilla gorilla* | 2.04 | 0.49 | 0.39 | 0.3 | 3 | 2 |
| MCZ23160 | M | *Gorilla gorilla* | 1.44 | 0.69 | 0.04 | 0.49 | 2 | 2 |
| MCZ23162 | M | *Gorilla gorilla* | 1.91 | 0.52 | 0.24 | 0.36 | 1 | 1 |
| MCZ17684 | F | *Gorilla gorilla* | 2.35 | 0.43 | 0.35 | 0.44 | 3 | 2 |
| MCZ37264 | F | *Gorilla gorilla* | 4.37 | 0.23 | 0.36 | 0.43 | 2 | 2 |
| MCZ38326 | F | *Gorilla gorilla* | 2.4 | 0.42 | 0.17 | 0.62 | 2 | 2 |
| MCZ29047 | F | *Gorilla gorilla* | 2.18 | 0.46 | 0.38 | 0.49 | 3 | 2 |
| MCZ26850 | F | *Gorilla gorilla* | 1.92 | 0.52 | 0.26 | 0.54 | 3 | 2 |
| MCZ37362 | M | *Pongo pygmaeus* | 1.98 | 0.5 | 0.35 | 0.41 | 3 | 2 |
| MCZ37365 | F | *Pongo pygmaeus* | 1.77 | 0.57 | 0.23 | 0.52 | 3 | 1 |
| MCZ37363 | F | *Pongo pygmaeus* | 3.01 | 0.33 | 0.52 | 0.42 | 3 | 3 |
| MCZ50958 | F | *Pongo pygmaeus* | 3.48 | 0.29 | 0.37 | 0.56 | 3 | 3 |
| MCZ50960 | F | *Pongo pygmaeus* | 1.46 | 0.69 | 0.12 | 0.49 | 1 | 3 |
| MCZ41541 | M | *Hylobates lar* | 2.11 | 0.48 | 0.19 | 0.31 | 2 | 2 |
| MCZ41529 | M | *Hylobates lar* | 1.63 | 0.62 | 0.07 | 0.38 | 2 | 3 |
| MCZ41534 | M | *Hylobates lar* | 3.28 | 0.31 | 0.25 | 0.45 | 2 | 2 |
| MCZ41532 | M | *Hylobates lar* | 3.18 | 0.32 | 0.08 | 0.42 | 2 | 2 |
| MCZ41531 | M | *Hylobates lar* | 5.73 | 0.18 | 0.38 | 0.33 | 2 | 1 |
| MCZ41540 | F | *Hylobates lar* | 2.97 | 0.34 | 0.42 | 0.35 | 2 | 2 |
| MCZ41543 | F | *Hylobates lar* | 2.83 | 0.35 | 0.3 | 0.45 | 2 | 2 |
