# Supplementary material for: Trabecular Architecture of the Manual Elements Reflects Locomotor Patterns in Primates
Source: PLoS One. 2015 Mar 20;10(3):e0120436. doi: 10.1371/journal.pone.0120436 (PMC4368714; doi:10.1371/journal.pone.0120436)
Supplement: S5 Table — See legend of S1 Table for code definitions. (DOCX) [file pone.0120436.s005.docx]

Table S5. Star Volume Distribution (SVD) variables for the Metacarpal Heads (Digit 3)

| **ID NUMBER** | **SEX** | **GENUS** | **SVD DA** | **SVD I** | **SVD E** | **SVD BV/TV** | **Shape Code** | **Orientation Code** |
| --- | --- | --- | --- | --- | --- | --- | --- | --- |
| MCZ19187 | M | *Pan Troglodytes* |  |  |  |  |  |  |
| MCZ20041 | M | *Pan Troglodytes* | 0.41 | 2.55 | 0.39 | 0.14 | 2 | 2 |
| MCZ23163 | M | *Pan Troglodytes* | 0.34 | 4.28 | 0.23 | 0.38 | 2 | 3 |
| MCZ48686 | M | *Pan Troglodytes* |  |  |  |  |  |  |
| MCZ26847 | F | *Pan Troglodytes* | 0.5 | 1.89 | 0.54 | 0.42 | 3 | 2 |
| MCZ26849 | F | *Pan Troglodytes* | 0.52 | 1.55 | 0.64 | 0.19 | 3 | 2 |
| MCZ15312 | F | *Pan Troglodytes* | 0.52 | 1.51 | 0.66 | 0.27 | 3 | 1 |
| APC232 | F | *Pan Troglodytes* | 0.39 | 5.24 | 0.19 | 0.16 | 2 | 2 |
| APC77 | M | *Macaca mulatta* | 0.23 | 5.06 | 0.2 | 0.3 | 2 | 3 |
| APC88 | M | *Macaca mulatta* | 0.23 | 3.1 | 0.32 | 0.48 | 2 | 3 |
| APC224 | M | *Macaca mulatta* | 0.25 | 4.17 | 0.24 | 0.23 | 2 | 2 |
| APC279 | M | *Macaca mulatta* | 0.27 | 4.48 | 0.22 | 0.3 | 2 | 2 |
| APC286 | M | *Macaca mulatta* | 0.3 | 3.5 | 0.29 | 0.47 | 2 | 3 |
| MCZ20039 | M | *Gorilla gorilla* | 0.47 | 1.92 | 0.52 | 0.42 | 3 | 2 |
| MCZ29048 | M | *Gorilla gorilla* | 0.3 | 11.03 | 0.09 | 0.15 | 2 | 2 |
| MCZ29049 | M | *Gorilla gorilla* |  |  |  |  |  |  |
| MCZ23160 | M | *Gorilla gorilla* | 0.34 | 5.57 | 0.18 | 0.32 | 2 | 2 |
| MCZ23162 | M | *Gorilla gorilla* | 0.2 | 13.2 | 0.08 | 0.47 | 2 | 2 |
| MCZ17684 | F | *Gorilla gorilla* | 0.38 | 5.99 | 0.17 | 0.36 | 2 | 2 |
| MCZ37264 | F | *Gorilla gorilla* | 0.42 | 5.29 | 0.19 | 0.48 | 2 | 3 |
| MCZ38326 | F | *Gorilla gorilla* | 0.43 | 6.34 | 0.16 | 0.29 | 2 | 2 |
| MCZ29047 | F | *Gorilla gorilla* | 0.4 | 5.2 | 0.19 | 0.33 | 2 | 3 |
| MCZ26850 | F | *Gorilla gorilla* | 0.4 | 4.64 | 0.22 | 0.35 | 2 | 2 |
| MCZ37362 | M | *Pongo pygmaeus* | 0.4 | 1.74 | 0.58 | 0.38 | 3 | 3 |
| MCZ37365 | F | *Pongo pygmaeus* | 0.44 | 3.01 | 0.33 | 0.58 | 2 | 3 |
| MCZ37363 | F | *Pongo pygmaeus* | 0.33 | 2.99 | 0.33 | 0.38 | 2 | 3 |
| MCZ50958 | F | *Pongo pygmaeus* | 0.41 | 1.8 | 0.56 | 0.35 | 3 | 3 |
| MCZ50960 | F | *Pongo pygmaeus* | 0.37 | 1.94 | 0.51 | 0.39 | 3 | 3 |
| MCZ41541 | M | *Hylobates lar* | 0.43 | 4.12 | 0.24 | 0.43 | 2 | 3 |
| MCZ41529 | M | *Hylobates lar* | 0.38 | 2.43 | 0.41 | 0.17 | 2 | 3 |
| MCZ41534 | M | *Hylobates lar* | 0.39 | 2.84 | 0.35 | 0.47 | 2 | 3 |
| MCZ41532 | M | *Hylobates lar* | 0.45 | 2.29 | 0.44 | 0.37 | 3 | 3 |
| MCZ41531 | M | *Hylobates lar* | 0.31 | 4.56 | 0.22 | 0.54 | 3 | 3 |
| MCZ41540 | F | *Hylobates lar* | 0.32 | 5.96 | 0.17 | 0.6 | 3 | 3 |
| MCZ41543 | F | *Hylobates lar* |  |  |  |  |  |  |
